# Estrogen Receptor beta (ERβ) Regulation of Lipid Homeostasis—Does Sex Matter?

**DOI:** 10.3390/metabo10030116

**Published:** 2020-03-20

**Authors:** Christina Savva, Marion Korach-André

**Affiliations:** 1 Department of Medicine, Metabolism Unit and KI/AZ Integrated Cardio Metabolic Center (ICMC), Metabolism and Karolinska Institutet at Karolinska University Hospital Huddinge, SE-141 86 Stockholm, Sweden; Christina.savva@ki.se; 2 Clinical Department of Endocrinology Diabetes, Karolinska University Hospital Huddinge, SE-141 86 Stockholm, Sweden

**Keywords:** estrogen receptor beta, lipid metabolism, sex, white adipose tissue, brown adipose tissue, liver

## Abstract

In this communication, we aim to summarize the role of estrogen receptor beta (ERβ) in lipid metabolism in the main metabolic organs with a special focus on sex differences. The action of ERβ is tissue-specific and acts in a sex-dependent manner, emphasizing the necessity of developing sex- and tissue-selective targeting drugs in the future.

## 1. Introduction

There is substantial evidence that females and males differ in their basic metabolic physiology and in their susceptibility to developing obesity-associated metabolic dysfunctions including insulin resistance, low-grade inflammation and fatty liver diseases. Interestingly, the response to excessive food intake is sex-dependent, e.g., obesity is more prevalent in women than in men, as type 2 diabetes (T2D) is more likely to be associated with obesity in men or postmenopausal women rather than in young, fertile women [1]. These changes over the lifespan or as a function of lifestyle make these sex differences even more difficult to treat. A better understanding of the sex differences in body composition would facilitate the anticipation of these changes and prevent the development of associated metabolic complications. Importantly, these differences in metabolic adaptations to disease infer that one sex has a specific attribute that protects them from disease. If that trait can be modulated, either directly or by modifying its downstream pathways, then disease development and/or progression may be tempered.

17β-Estradiol (estrogen, E_2_) binds to both of the estrogen receptors (ERα and ERβ) as well as the membrane-bound G-protein-coupled ER (GPER1). ERα and ERβ are the main receptors that mediate the genomic action of E_2_; while GPER1 is best known for its ability to regulate cell signaling, it may also synchronize gene expression [2]. E_2_ treatment reduces adiposity in both sexes and improves metabolic adaptation to obesity through the activation of both ERs [3,4]. However, it also mediates cell proliferation through activation of ERα present in target tissues and can thus contribute to malignant growth in these tissues. These detrimental effects render the use of E_2_- and/or ERα-selective agonists as a treatment for obesity difficult, whereas ERβ is thought to counteract these activities [5]. Recently, selective activation of ERβ has demonstrated beneficial outcomes on metabolic control in obesity [6,7,8,9], probably through feedback mechanisms, since ERβ is expressed at very low levels in metabolic tissues including the liver. In this review, we aim to summarize the current understanding of the role of ERβ in the regulation of lipid homeostasis, with a special focus on sex differences in obesity and associated metabolic dysfunctions.

## 2. ERα Versus ERβ, Laboratory Mouse and Ligands in Metabolic Studies

It has been demonstrated that estrogens are involved in the regulation of metabolic processes by investigating the actions of ERα and ERβ using appropriate models lacking either ERα (αERKO) [10] or ERβ (βERKO) [11,12]. Aromatase knockout (ArKO) [13] or ovariectomized (OVX) [14] mice are also models that are frequently used in research on estrogen receptor signaling due to loss of, or reduced, circulating estrogens. Moreover, the activation of ERs by synthetic ligands/agonists is another approach used in endocrinology studies in both males and females that may better reflect human physiology. A large number of ERβ-selective ligands including DPN [11], WAY200070 [15], β-LGNDs [8,12], LY3201 [10] and DIP [6,9] have been used to further investigate the role of ERβ in metabolic homeostasis.

## 3. ERβ in Visceral and Subcutaneous Adipose Tissue

It is well established that sex hormones are a key driving factor behind the sex differences in the regulation of adiposity and fat distribution; however, the mechanism is still unclear. Men and postmenopausal women, in general, have less total body fat and a higher accumulation of visceral adipose tissue (VAT) characterized as the “male, apple shape fat distribution phenotype”. Premenopausal women accumulate more gluteal and subcutaneous adipose tissue (SAT) characterized as the “female, pear shape fat distribution phenotype” [16,17,18,19,20]. The decreased circulating hormones in postmenopausal women may explain the increased visceral obesity that is highly correlated with metabolic complications. Interestingly, hormone replacement therapy (HRT) has been considered as a method for reversing this phenomenon [21]. Nevertheless, the timing of HRT initiation has been shown to play a crucial role in the beneficial effect of therapy [22].

Both human and rodent adipose tissue express ERα and ERβ [14,23,24,25]. In humans, both ERs exist in SAT and VAT in both genders with no gender differences in the expression level of *Erα*, while *Erβ* is expressed to a greater degree in women in both SAT and VAT compared to men but is certainly lower than *Erα* [24]. *Erα* and *Erβ* gene expression is increased in adipocytes in SAT in premenopausal women treated with E_2_ in vitro. However, in adipocytes from men, only the *Erα* subtype was increased by E_2_ in both fat pads [24]. In contrast, Anwar et al. showed that the protein level of ERα is decreased in postmenopausal female SAT cells treated with E_2_ [25]. Interestingly, in postmenopausal women, *Erα* expression level is unchanged in SAT, as opposed to *Erβ* that shows increased expression compared to premenopausal women [26]. The differences in the expression levels of the two subtypes creates an unstable ratio of ERα/ERβ, which could account for the different biological activity of estrogens in men and women. A correlation between obesity and the ratio of ERα and ERβ in SAT and VAT was found [27]. It is possible that the differences in expression levels of ERα and ERβ in the various fat pads could explain the shift of SAT and VAT between the pre- and postmenopausal women states.

Recent studies using female and male animal models have highlighted the key role of ERβ in the regulation of white adipose tissue (WAT) between genders. Female and male mice lacking ERβ (ERβKO) increased their body weight and fat mass [11,12]. A recent study performed on ovariectomized versus intact 1-year-old mice claimed that ERβ, but not ERα, may be required to bring about beneficial metabolic outcomes after ovariectomy [28]. Administration of ERβ-selective ligand (β-LGND2) provokes a reduction in body weight and a significant loss of total fat mass in wild-type (WT) mice on a high-fat diet (HFD), without altering food consumption, but not in ERβKO male mice [12]. HFD male mice treated with the ERβ-selective ligand (β-LGND1/β-LGND2) exhibited decreased weight gain and total fat accumulation in WAT. In the same study, ovariectomized female mice increased their fat mass, and the administration of β-LGND2 reversed this effect [8]. Conversely, in vivo magnetic resonance imaging measurement in the same animals, before and after administration of the drug, revealed that ERβ-selective ligand (DIP) administration reduced total body fat accumulation in HFD female young mice by reducing de novo lipogenesis [6], but this was not observed in HFD male mice [9]. Most interestingly, DIP administration in HFD males caused a remodeling of the fat towards a feminized distribution (i.e., increased SAT and unchanged VAT) [9]. In a recent study, we showed that sex-specific fatty acid and triglyceride (TG) pathways exist in both adipose depot VAT and SAT in *ob/ob* mouse fed a control diet [20], with males synthetizing more C18:2*n-6 trans* fatty acid associated with inflammation, and more of the long-chain TGs in VAT compared to females. These differences could be the consequence of the different genetic basis of fat distribution between the sexes [29]. In addition, female mice are more responsive to recruitment of brown adipocytes in VAT than male mice, probably due to the higher level of estrogen-dependent sympathetic innervation [30]. One possible mechanism that could be behind the actions of ERβ in lipid homeostasis is the cross-talk between ERβ and PPARγ, where ERβ inhibits the ligand-mediated PPARγ activity that leads to reduced adipogenesis [8,11]. Another possibility is the cross-talk with hepatic stellate cells, where ERβ but not ERα is expressed [31], as has been suggested by several studies [32,33,34].

## 4. ERβ Function in Hepatic Lipid Metabolism

The role of ERα in liver metabolism homeostasis is well established. Studies using either ERαKO mice of both sexes or ERα-specific ligand show that the presence of ERα has beneficial effects on liver metabolism, glucose tolerance and hepatic insulin sensitivity [35,36]. However, the adverse effect of ERα activation on uterus growth and breast cancer development has limited the use of ERα agonist as a treatment for liver metabolic disorders. The role of ERβ in liver metabolism including insulin sensitivity is less clear, especially as ERβ is minimally expressed in hepatocytes [37,38]. Nevertheless, activation of ERβ by selective agonists has anti-obesogenic effects, prevents hepatic lipid accumulation and reduces lipogenic gene expression levels [8,39]. Conversely, ERβKO mice showed decreased TG accumulation and improved whole-body insulin sensitivity and glucose tolerance [11], while ERβ activation improved insulin sensitivity in obese female and male mice [6,9,12,40]. In recent publications, ERβ activation by the selective synthetic ligand DIP resulted in a reduction of lipid accumulation in the liver in HFD female mice only, by means of both a reduction in de novo lipogenesis and increased lipid breakdown, demonstrated by a deuterium labelling method. Interestingly, DIP treatment in HFD-fed mice provokes a remodeling of triglyceride composition with a reduction in the fraction of saturated lipids and an increase in the fraction of unsaturated lipids in both genders, as has been demonstrated in vivo by magnetic resonance spectroscopy, using the animal as its own control [6,9]. Importantly, both ERβ and ERα are key regulators of the phospholipid and fatty acid pathways in female and male *ob/ob* mouse liver, by controlling the transcriptional activity of key genes in these pathways [20]. Male livers synthesized more long-chain triglycerides and phospholipids containing lipotoxic fatty acids than did female livers, which may contribute to the sexual dimorphism in the metabolic adaptation to obesity towards more metaflammation in males than in females [20]. Controversially, in some studies ERβ failed to show positive regulation of insulin-mediated glucose disposal and insulin signaling in both sexes [35]. Indeed, liver cells express very little the ERβ subtype compared to ERα; therefore, the effects observed by the activation of ERβ by a ligand might result from a feedback loop or crosstalk from other tissues. Hepatic stellate cells contain ERβ but not ERα, and it has been suggested that the ERβ-selective agonist DPN ameliorates liver cirrhosis in rat through the inhibition of hepatic stellate cell proliferation [41]. Taken together, these data suggest that ERβ would represent a promising target as an anti-obesogenic, anti-fatty liver disease treatment; however, more studies should be conducted in order to clarify the role of ERβ on liver homeostasis.

## 5. ERβ in Brown and Beige Adipose Tissue

The ability of brown adipose tissue (BAT) to oxidize lipids and generate heat through the mitochondrial uncoupling protein (UCP1) is unique [12] and has led to interest in targeting BAT to combat obesity. Several studies have observed differences in BAT activity between the sexes, with females having higher metabolically active BAT compared to males [42,43]. Both ERs are expressed in human fetal BAT, suggesting a key role for the two subtypes in BAT development, even though ERα is the predominant one. However, ERβ was only present in mature brown adipocytes, which supports the theory that the differentiation process to brown adipocytes probably occurs through ERα [44]. BAT from female mice is enriched in arachidonic and stearic acid phospholipid and depleted in triglycerides compared to males. It has been suggested that these sex specificities will influence mitochondrial membranes and other organelles, which will in turn affect tissue function [45]. Cold exposure and high-fat diet intake induce browning of adipose tissue [12,46]. In old obese WT and ERαKO female mice, but not young female and male WT mice, the administration of ERβ ligand (LY3201) caused browning of SAT through the increased expression of UCP1. Additionally, it is interesting to note that males had lower expression of ERβ in SAT compared to females, which could explain the absence of the effect of the treatment in males [10]. In female mice, ERβ activation by DIP enhances BAT activity by inducing the expression of UCP1 [6]. Conversely, in males, the accumulation of larger lipid droplets in BAT was observed after DIP treatment, together with the generation of heat measured in vivo by comprehensive laboratory animal monitoring system (CLAMS), that could result from browning sites in the VAT [9]. In another study, ERβ-selective agonist (β-LGND1/2) given to HFD male mice prevented body weight gain and fat storage by inducing browning of white adipose tissue [8,12]. However, ERβ was shown to be more potent at suppressing adipose-derived stem cell brown adipose tissue differentiation, from male mice, through decreased expression of *Ucp1*, *Pgc1α* and *Pparγ* genes [47]. Therefore, inducing browning in white adipose tissue through ERβ activation could be of clinical relevance to tackle obesity.

## 6. Conclusions

There is no controversy about the fact that estrogens have important physiological actions in the regulation of lipid homeostasis in both females and males. The expression of both ERβ and ERα fluctuates in various metabolic tissues, which complicates our current understanding of their distinct role in the regulation of energy and lipid homeostasis. More recently, extensive research has demonstrated beneficial metabolic outcomes of ERβ activation and has defined a central role for ERβ in metabolic control. However, further research is needed to elucidate the role of ERβ in lipid homeostasis. Targeting ERβ in order to tackle metabolic disorders associated with obesity without inducing the side effects of ERα activation could be a potential solution. However, numerous studies have demonstrated that ERβ action is tissue-specific and acts in a sex-dependent manner, highlighting the need to further develop sex- and tissue-selective targeting drugs in the future.

## References

[1] Regitz-Zagrosek V., Lehmkuhl E., Weickert M.O. (2006). Gender differences in the metabolic syndrome and their role for cardiovascular disease. Clin. Res. Cardiol..

[2] Prossnitz E.R., Barton M. (2011). The G-protein-coupled estrogen receptor GPER in health and disease. Nat. Rev. Endocrinol..

[3] Lizcano F., Guzmán G. (2014). Estrogen Deficiency and the Origin of Obesity during Menopause. BioMed Res. Int..

[4] Rubinow K.B. (2017). Estrogens and Body Weight Regulation in Men. Adv. Exp. Med. Biol..

[5] Lindberg M.K., Movérare S., Skrtic S., Gao H., Dahlman-Wright K., Gustafsson J.-Å., Ohlsson C. (2003). Estrogen Receptor (ER)-β Reduces ERα-Regulated Gene Transcription, Supporting a “Ying Yang” Relationship between ERα and ERβ in Mice. Mol. Endocrinol..

[6] González-Granillo M., Savva C., Li X., Fitch M., Pedrelli M., Hellerstein M., Parini P., Korach-André M., Gustafsson J.-Å. (2019). ERβ activation in obesity improves whole body metabolism via adipose tissue function and enhanced mitochondria biogenesis. Mol. Cell. Endocrinol..

[7] Liang Y.-Q., Akishita M., Kim S., Ako J., Hashimoto M., Iijima K., Ohike Y., Watanabe T., Sudoh N., Toba K. (2002). Estrogen receptor ß is involved in the anorectic action of estrogen. Int. J. Obes..

[8] Yepuru M., Eswaraka J., Kearbey J.D., Barrett C.M., Raghow S., Veverka K.A., Miller D.D., Dalton J.T., Narayanan R. (2010). Estrogen Receptor-β-selective Ligands Alleviate High-fat Diet- and Ovariectomy-induced Obesity in Mice. J. Boil. Chem..

[9] González-Granillo M., Savva C., Li X., Laskar M.G., Angelin B., Gustafsson J.-Å., Korach-André M. (2020). Selective estrogen receptor (ER)β activation provokes a redistribution of fat mass and modifies hepatic triglyceride composition in obese male mice. Mol. Cell. Endocrinol..

[10] Miao Y.-F., Su W., Dai Y.-B., Wu W.-F., Huang B., Barros R.P.A., Nguyen H., Maneix L., Guan Y.-F., Warner M. (2016). An ERβ agonist induces browning of subcutaneous abdominal fat pad in obese female mice. Sci. Rep..

[11] Foryst-Ludwig A., Clemenz M., Hohmann S., Hartge M., Sprang C., Frost N., Krikov M., Bhanot S., Barros R., Morani A. (2008). Metabolic Actions of Estrogen Receptor Beta (ERβ) are Mediated by a Negative Cross-Talk with PPARγ. PLoS Genet..

[12] Ponnusamy S., Tran Q.T., Harvey I., Smallwood H.S., Thiyagarajan T., Banerjee S., Johnson D.L., Dalton J.T., Sullivan R., Miller D.D. (2016). Pharmacologic activation of estrogen receptor α increases mitochondrial function, energy expenditure, and brown adipose tissue. FASEB J..

[13] Jones M., Thorburn A.W., Britt K., Hewitt K.N., Misso M.L., Wreford N.G., Proietto J., Oz O.K., Leury B., Robertson K.M. (2001). Aromatase-deficient (ArKO) mice accumulate excess adipose tissue. J. Steroid Biochem. Mol. Boil..

[14] Naaz A., Zakroczymski M., Heine P., Taylor J., Saunders P., Lubahn D., Cooke P.S. (2002). Effect of Ovariectomy on Adipose Tissue of Mice in the Absence of Estrogen Receptor Alpha (ERα): A Potential Role for Estrogen Receptor Beta (ERβ). Horm. Metab. Res..

[15] Magdalena P.A., Ropero A.B., García-Arévalo M., Soriano S., Quesada I., Muhammed S.J., Salehi A., Gustafsson J.-A., Nadal Á. (2013). Antidiabetic Actions of an Estrogen Receptor β Selective Agonist. Diabetes.

[16] Demerath E., Sun S.S., Rogers N., Lee M., Reed D., Choh A.C., Couch W., Czerwinski S.A., Chumlea W.C., Siervogel R.M. (2007). Anatomical Patterning of Visceral Adipose Tissue: Race, Sex, and Age Variation. Obesity.

[17] Macotela Y., Boucher J., Tran T.T., Kahn C.R. (2009). Sex and Depot Differences in Adipocyte Insulin Sensitivity and Glucose Metabolism. Diabetes.

[18] Grove K.L., Fried S.K., Greenberg A.S., Xiao X., Clegg D.J. (2010). A microarray analysis of sexual dimorphism of adipose tissues in high-fat-diet-induced obese mice. Int. J. Obes..

[19] Karastergiou K., Smith S.R., Greenberg A.S., Fried S.K. (2012). Sex differences in human adipose tissues – the biology of pear shape. Boil. Sex Differ..

[20] González-Granillo M., Helguero L., Alves E., Archer A., Savva C., Pedrelli M., Ahmed O., Li X., Domingues M.R., Parini P. (2019). Sex-specific lipid molecular signatures in obesity-associated metabolic dysfunctions revealed by lipidomic characterization in ob/ob mouse. Boil. Sex Differ..

[21] Gambacciani M., Ciaponi M., Cappagli B., De Simone L., Orlandi R., Genazzani A.R. (2001). Prospective evaluation of body weight and body fat distribution in early postmenopausal women with and without hormonal replacement therapy. Maturitas.

[22] Lobo R.A. (2016). Hormone-replacement therapy: Current thinking. Nat. Rev. Endocrinol..

[23] Pedersen S.B., Bruun J., Hube F., Kristensen K., Hauner H., Richelsen B., Richelsen B. (2001). Demonstration of estrogen receptor subtypes α and β in human adipose tissue: Influences of adipose cell differentiation and fat depot localization. Mol. Cell. Endocrinol..

[24] Dieudonne M.N., Leneveu M.C., Giudicelli Y., Pecquery R. (2004). Evidence for functional estrogen receptors α and β in human adipose cells: Regional specificities and regulation by estrogens. Am. J. Physiol. Physiol..

[25] Anwar A., McTernan P.G., Anderson L.A., Askaa J., Moody C.G., Barnett A.H., Eggo M.C., Kumar S. (2001). Site-specific regulation of oestrogen receptor-α and -β by oestradiol in human adipose tissue. Diabetes Obes. Metab..

[26] McInnes K.J., Andersson T.C., Simonyte K., Söderström I., Mattsson C., Seckl J.R., Olsson T. (2012). Association of 11β-Hydroxysteroid Dehydrogenase Type 1 Expression and Activity with Estrogen Receptor β in Adipose Tissue from Postmenopausal Women. Menopause.

[27] Shin J.-H., Hur J.-Y., Seo H.S., Jeong Y.-A., Lee J.K., Oh M.-J., Kim T., Saw H.S., Kim S.H. (2007). The ratio of estrogen receptor α to estrogen receptor β in adipose tissue is associated with leptin production and obesity. Steroids.

[28] Zidon T.M., Padilla J., Fritsche K.L., Welly R.J., McCabe L.T., Stricklin O.E., Frank A.P., Park Y.M., Clegg D.J., Lubahn D. (2020). Effects of ERbeta and ERalpha on OVX-induced changes in adiposity and insulin resistance. J. Endocrinol..

[29] Heid I.M., Jackson A.U., Randall J., Winkler T.W., Qi L., Steinthorsdottir V., Thorleifsson G., Zillikens M.C., Speliotes E.K., Magi R. (2010). Meta-analysis identifies 13 new loci associated with waist-hip ratio and reveals sexual dimorphism in the genetic basis of fat distribution. Nat. Genet..

[30] Kim S.-N., Jung Y.-S., Kwon H.-J., Seong J.K., Granneman J.G., Lee Y.-H. (2016). Sex differences in sympathetic innervation and browning of white adipose tissue of mice. Boil. Sex Differ..

[31] Zhou Y., Shimizu I., Lu G., Itonaga M., Okamura Y., Shono M., Honda H., Inoue S., Muramatsu M., Ito S. (2001). Hepatic Stellate Cells Contain the Functional Estrogen Receptor β but Not the Estrogen Receptor α in Male and Female Rats. Biochem. Biophys. Res. Commun..

[32] She H., Xiong S., Hazra S., Tsukamoto H. (2004). Adipogenic Transcriptional Regulation of Hepatic Stellate Cells. J. Boil. Chem..

[33] Shimizu I., Kohno N., Tamaki K., Shono M., Huang H.-W., He J.-H., Yao D.-F. (2007). Female hepatology: Favorable role of estrogen in chronic liver disease with hepatitis B virus infection. World J. Gastroenterol..

[34] Shimizu I., Mizobuchi Y., Yasuda M., Shiba M., Ma Y.R., Horie T., Liu F., Ito S. (1999). Inhibitory effect of oestradiol on activation of rat hepatic stellate cells in vivo and in vitro. Gut.

[35] Bryzgalova G., Gao H., Ahrén B., Zierath J.R., Galuska D., Steiler T., Dahlman-Wright K., Nilsson S., Gustafsson J.-A., Efendic S. (2006). Evidence that oestrogen receptor-α plays an important role in the regulation of glucose homeostasis in mice: Insulin sensitivity in the liver. Diabetologia.

[36] Heine P.A., Taylor J.A., Iwamoto G.A., Lubahn D.B., Cooke P.S. (2000). Increased adipose tissue in male and female estrogen receptor-alpha knockout mice. Proc. Natl. Acad. Sci. USA.

[37] Ribas V., Nguyen M.T.A., Henstridge D.C., Nguyen A.-K., Beaven S.W., Watt M.J., Hevener A.L. (2009). Impaired oxidative metabolism and inflammation are associated with insulin resistance in ERalpha-deficient mice. Am. J. Physiol. Metab..

[38] Chow J.D., Jones M.E., Prelle K., Simpson E.R., Boon W.C. (2011). A selective estrogen receptor alpha agonist ameliorates hepatic steatosis in the male aromatase knockout mouse. J. Endocrinol..

[39] Shin E.S., Lee H.H., Cho S.Y., Park H.W., Lee S.J., Lee T.R. (2007). Genistein Downregulates SREBP-1 Regulated Gene Expression by Inhibiting Site-1 Protease Expression in HepG2 Cells. J. Nutr..

[40] Ponnusamy K.E., Somerville L., McCalden R.W., Marsh J., Vasarhelyi E.M. (2019). Revision Rates and Functional Outcome Scores for Severely, Morbidly, and Super-Obese Patients Undergoing Primary Total Hip Arthroplasty. JBJS Rev..

[41] Zhang B., Zhang C.-G., Ji L., Zhao G., Wu Z. (2018). Estrogen receptorβselective agonist ameliorates liver cirrhosis in rats by inhibiting the activation and proliferation of hepatic stellate cells. J. Gastroenterol. Hepatol..

[42] Poirier P., Giles T.D., Bray G.A., Hong Y., Stern J.S., Pi-Sunyer F.X., Eckel R.H. (2006). Obesity and Cardiovascular Disease: Pathophysiology, Evaluation, and Effect of Weight Loss. Circulation.

[43] Choi D.K., Oh T.S., Choi J.-W., Mukherjee R., Wang X., Liu H., Yun J.W. (2011). Gender Difference in Proteome of Brown Adipose Tissues between Male and Female Rats Exposed to a High Fat Diet. Cell. Physiol. Biochem..

[44] Velickovic K., Cvoro A., Srdić B., Stokić E., Markelić M., Golic I., Otasevic V., Stancic A., Jankovic A., Vučetić M. (2014). Expression and Subcellular Localization of Estrogen Receptors α and β in Human Fetal Brown Adipose Tissue. J. Clin. Endocrinol. Metab..

[45] Hoene M., Li J., Häring H.-U., Weigert C., Xu G., Lehmann R. (2014). The lipid profile of brown adipose tissue is sex-specific in mice. Biochim. Biophys. Acta (BBA) Mol. Cell Boil. Lipids.

[46] García-Ruiz E., Reynés B., Diaz-Rua R., Ceresi E., Oliver P., Palou A. (2015). The intake of high-fat diets induces the acquisition of brown adipocyte gene expression features in white adipose tissue. Int. J. Obes..

[47] Zhang W., Schmull S., Du M., Liu J., Lu Z., Zhu H., Xue S., Lian F. (2016). Estrogen Receptor α and β in Mouse: Adipose-Derived Stem Cell Proliferation, Migration, and Brown Adipogenesis In Vitro. Cell. Physiol. Biochem..

